# Species-Dependent Metabolic Response to Lipid Mixtures in Wine Yeasts

**DOI:** 10.3389/fmicb.2022.823581

**Published:** 2022-05-23

**Authors:** Lethiwe L. Mbuyane, Florian F. Bauer, Audrey Bloem, Carole Camarasa, Anne Ortiz-Julien, Benoit Divol

**Affiliations:** ^1^Department of Viticulture and Oenology, South African Grape and Wine Research Institute, Stellenbosch University, Stellenbosch, South Africa; ^2^UMR SPO, INRA, SupAgroM, Université de Montpellier, Montpellier, France; ^3^Lallemand SAS, Blagnac, France

**Keywords:** fatty acids, ergosterol, wine, yeasts, acetic acid, metabolic flux, acetyl-CoA

## Abstract

Lipids are essential energy storage compounds and are the core structural elements of all biological membranes. During wine alcoholic fermentation, the ability of yeasts to adjust the lipid composition of the plasma membrane partly determines their ability to cope with various fermentation-related stresses, including elevated levels of ethanol and the presence of weak acids. In addition, the lipid composition of grape juice also impacts the production of many wine-relevant aromatic compounds. Several studies have evaluated the impact of lipids and of their metabolism on fermentation performance and aroma production in the dominant wine yeast *Saccharomyces cerevisiae*, but limited information is available on other yeast species. Thus, the aim of this study was to evaluate the influence of specific fatty acid and sterol mixtures on various non-*Saccharomyces* yeast fermentation rates and the production of primary fermentation metabolites. The data show that the response to different lipid mixtures is species-dependent. For *Metschnikowia pulcherrima*, a slight increase in carbon dioxide production was observed in media enriched with unsaturated fatty acids whereas *Kluyveromyces marxianus* fermented significantly better in synthetic media containing a higher concentration of polyunsaturated fatty acids than monounsaturated fatty acids. *Torulaspora delbrueckii* fermentation rate increased in media supplemented with lipids present at an equimolar concentration. The data indicate that these different responses may be linked to variations in the lipid profile of these yeasts and divergent metabolic activities, in particular the regulation of acetyl-CoA metabolism. Finally, the results suggest that the yeast metabolic footprint and ultimately the wine organoleptic properties could be optimized *via* species-specific lipid adjustments.

## Introduction

Non-*Saccharomyces* yeasts have become valuable in the wine industry due to their potential to diversify wine flavor profiles and/or address specific (bio)technological challenges (Capozzi et al., 2015; Padilla et al., 2016; Rossouw and Bauer, 2016; Furlani et al., 2017). Despite these benefits, these yeasts cannot fully replace the common wine yeast *Saccharomyces cerevisiae* as they fail to ferment to completion. Multi-starter or sequential fermentations with *S. cerevisiae* are therefore required to ensure the complete utilization of all sugars (Bely et al., 2008; Gobbi et al., 2013; Taillandier et al., 2014). Indeed, a successful fermentation requires a yeast strain that is resistant to osmotic pressure and to the accumulation of ethanol and other toxic molecules (weak acids, antimicrobial peptides, etc.) as well as oxygen limitation (Bauer and Pretorius, 2000; Rossignol et al., 2003; Shima and Takagi, 2009; Tronchoni et al., 2017). The ability of yeast species to respond to these constraints determines the extent of their survival and overall fermentation performance (Mannazzu et al., 2008; Rodríguez-Porrata et al., 2011; Oro et al., 2014; Curiel et al., 2017).

Lipids play a major role in mediating yeast stress response. Indeed, lipids such as sterols, phospholipids and sphingolipids are essential components of the yeast plasma membrane. Adjustments in the lipid composition of this relatively impermeable barrier enables the yeast cell to adjust to various fermentation conditions such as ethanol accumulation, low pH and supports the transport of specific molecules *via* a vast array of transport proteins (Van der Rest et al., 1995). Adjustments also include changes in the relative concentration of phospholipids, sterols and the saturation of fatty acids. However, the production of ergosterol, the main yeast sterol, and the desaturation of fatty acids require oxygen, an element in limited supply during self-anaerobic fermentations (Valero et al., 2002; Mannazzu et al., 2008; Archana et al., 2015).

During the early stages of wine alcoholic fermentation, yeasts synthesize ergosterol and unsaturated fatty acids when oxygen is present (Stukeys et al., 1990; Belviso et al., 2004; Liu et al., 2019). As fermentation progresses, conditions become rapidly anaerobic, and the lack of oxygen prevents the necessary modifications to the cell lipid composition to ensure yeast survival (Rosenfeld et al., 2003). Indeed, in *S. cerevisiae*, alcoholic fermentation in the absence of oxygen and exogenous lipids leads to an enrichment in saturated fatty acids, a decrease in the unsaturated fatty acid as well as sterol concentration. Furthermore, enhanced production of medium chain fatty acids is also observed under anaerobiosis, and these impacts may lead to reduced fermentation efficiency and sluggish or stuck fermentation (Bardi et al., 1999). An efficient fermentation therefore requires either the uptake of exogenous lipids or the presence of oxygen (Delfini et al., 1993; Burin et al., 2015; Houtman and Du Plessis, 2017).

Standard wine making practices impact lipid metabolism and composition. Indeed, yeast-derived fermentation additives (referred to as complex nutrients) containing significant lipid concentrations typically used in the rehydration of yeasts directly reinforce membrane integrity and support fermentative activity (Valero et al., 1998, 2001; Belviso et al., 2004; Varela et al., 2012; Ochando et al., 2017). Furthermore, aeration is used in winemaking to enhance or reactivate yeast viability or metabolism (Shekhawat et al., 2017). Indeed, the micro-oxygenation of fermenting grape must has been shown to increase yeast growth and performance during alcoholic fermentation due to the reactivation of lipid biosynthesis in *Torulaspora delbrueckii* and *S. cerevisiae* (Mauricio et al., 1998). Other authors showed that the cultivation of *Candida stellata*, *T. delbrueckii*, *Hanseniaspora uvarum*, *Hanseniaspora guilliermondii* and *Debaryomyces hansenii* under aerobic conditions impacts ethanol stress resistance (Pina et al., 2004). Oxygen sparging also influences the production of higher alcohols, medium fatty acids and esters during alcoholic fermentation (Fujii et al., 1997; Varela et al., 2012; Ochando et al., 2017). Although oxygen was observed to inhibit acetate ester synthesis in *S. cerevisiae via* the inhibition of the alcohol acetyl transferase *ATF1* and repressed ethyl ester production, an overall increase of ester production has been attributed to improved biomass formation (Mauricio et al., 1997; Varela et al., 2012; Ochando et al., 2017).

The addition of individual or defined mixtures of lipids including ergosterol and unsaturated fatty acids such as palmitoleic acid, oleic acid, linoleic and linolenic acid improves yeast performance during alcoholic fermentation (Ohta and Hayashida, 1983; Casu et al., 2016; Ochando et al., 2017; Fairbairn et al., 2019). However, the impact of these compounds on yeast survival and performance is concentration- and condition-dependent (You et al., 2003; Fairbairn et al., 2019; Liu et al., 2019). Furthermore, adjusting the lipid content *via* direct supplementation impacts the production of certain volatile compounds during alcoholic fermentation (Varela et al., 2012; Duan et al., 2015; Liu et al., 2018, 2020; Fairbairn et al., 2019). For instance, acetic acid production was observed to decrease after the addition of phytosterols and unsaturated fatty acids in *S. cerevisiae*, possibly due to a reduced need for acetyl-CoA, the main substrate of lipid metabolism. However, an increase in acetic acid was observed when higher amounts of phytosterols and unsaturated fatty acids were used and this was attributed to an increase in yeast biomass (Luparia et al., 2004; Ochando et al., 2017; Deroite et al., 2018). Moreover, certain species-specific responses have also been reported. Indeed, a previous study showed that the low levels of acetic acid observed in *M. pulcherrima* fermentations may result from a limitation of acetyl-CoA which also led to a reduction in medium chain fatty acids and their corresponding ethyl esters (Seguinot et al., 2020). However, the lipid composition of this yeast was not investigated. In this context, more studies are necessary to evaluate the utilization of lipids and their impact on yeast performance during alcoholic fermentation in non-*Saccharomyces* yeasts.

The aim of this study was to evaluate the impact of fatty acids and sterols on yeast fermentation performance and to determine whether the impact of these compounds on yeast performance is species-dependent. The impact of different lipid ratios and concentrations on yeast performance was evaluated in selected yeast strains belonging to three yeast species, namely *M. pulcherrima*, *T. delbrueckii* and *K. marxianus*.

## Materials and Methods

### Yeast Strains

The following yeast strains were used in the study: a *Metschnikowia pulcherrima* yeast strain (Strain A), a *Torulaspora delbrueckii* yeast strain (Strain B) and a *Saccharomyces cerevisiae* strain (Lalvin EC1118^®^) from Lallemand SA (Montreal, Canada) and *Kluyveromyces marxianus* (IWBT Y885). The latter yeast belongs to the yeast collection of the South African Grape and Wine Research Institute, Stellenbosch University, South Africa.

### Media

Fermentations were conducted in synthetic grape juice-like medium containing 100 g/l glucose, 100 g/l fructose, 2.5 g/l tartaric acid, 3 g/l malic acid, 0.2 g/l citric acid, 1.14 g/l potassium phosphate dibasic, 1.23 g/l magnesium sulfate heptahydrate, 0.44 g/l calcium chloride dihydrate, supplemented with vitamins, trace elements and 300 mg/l Yeast Assimilable Nitrogen (with amino acids and ammonium chloride) as shown in Table 1 (Bely et al., 1990; Henschke and Jiranek, 1993).

**TABLE 1:** Trace elements, vitamins and nitrogen stock (with amino acids and ammonium chloride) used to prepare grape juice-like synthetic medium.

Trace elements		Per liter	Vitamins	Per liter
**Trace elements and Vitamin stocks**				
	Manganese (III) chloride tetrahydrate (MnCl_2_, 4H_2_O)	0.02 g	Myo-inositol	10 g
	Zinc chloride (ZnCl_2_)	0.0135 g	Pyridoxine hydrochloride	0.2 g
	Iron (III) chloride (FeCl_2_)	0.003 g	Nicotinic acid	0.2 g
	Copper (III) chloride (CuCl_2_)	0.0015 g	Calcium pantothenate	0.1 g
	Boric acid (H_3_BO_3_)	0.0005 g	Thiamine hydrochloride	0.05 g
	Cobalt (II) nitrate hexahydrate [Co(NO_3_)_2_, 6H_2_O]	0.0030 g	PABA K	0.02 g
	Sodium molybdate dehydrate (NaMoO_4_, 2H_2_O)	0.0025 g	Riboflavin	0.02 g
	Potassium iodate (KlO_3_)	0.001 g	Biotin	0.0125 g
			Folic acid	0.02 g
**Nitrogen**				
	Tyrosine	1.83 g	Alanine	14.52 g
	Tryptophan	17.93 g	Valine	4.45 g
	Isoleucine	3.27 g	Methionine	3.14 g
	Aspartic acid	4.45 g	Phenylalanine	3.80 g
	Glutamic acid	12.04 g	Serine	7.85 g
	Arginine	37.34 g	Histidine	3.27 g
	Leucine	4.84 g	Lysine	1.70 g
	Threonine	7.59 g	Cysteine	1.31 g
	Glycine	1.83 g	Proline	61.26 g
	Asparagine	5.31 g	NH_4_Cl	46 g
	Glutamine	50.52 g		

### Fermentation Conditions

Freeze cultures were streaked onto Yeast Peptone Dextrose (YPD) Agar (Merck, Fontenay-sous-Bois, France) plates and incubated at 30°C for 48 h. A single colony was inoculated into 5 ml Yeast Nutrient Base (YNB) medium with 20 g/l sugar for 24 h. A 1 ml culture was then transferred into 100 ml YNB and incubated until the yeast populations reached late exponential growth under semi-aerobic conditions in an Erlenmeyer flask with 70% headspace. Yeast cultures were harvested, centrifuged at 4,193 *g* for 5 min, washed with saline solution (0.9% NaCl), inoculated into 250 ml synthetic medium containing myristic, palmitic, palmitoleic, stearic, oleic, linoleic, linolenic acid and ergosterol (Sigma, Saint-Quentin-Fallavier, France) in different combinations as shown in Table 2 and incubated at 25°C with agitation at 120 rpm. The fatty acids used in this study are commonly found in grape juice and the cell pellet. Ergosterol was used because it is the main sterol found in yeasts. Medium A contained equimolar concentrations of lipids, Medium B an equimolar lipid mixture with double the amount of unsaturated fatty acids and Medium C a grape juice-like lipid mixture based on previous studies (Yunoki et al., 2005; Tumanov et al., 2015). Fermentations were conducted in a 250-ml bottle with 10% headspace under anaerobic conditions. Dissolved oxygen was removed by sparging argon for 25 min prior to the addition of lipid mixtures. In order to evaluate the fatty acid and sterol composition of yeasts, self-evolving fermentations were performed in synthetic grape juice-like media without the addition of lipids. Yeasts were pre-cultured and inoculated as previously described and the fermentations were also incubated at 25°C.

**TABLE 2:** Lipid mixtures used for alcoholic fermentation in synthetic grape juice-like media.

Lipids	Medium A (mmol/L)	Medium B (mmol/L)	Medium C (mmol/L)
>Myristic acid (C14:0)	1.077	1.077	0.099
>Palmitic acid (C16:0)	1.077	1.077	2.491
>Palmitoleic acid (16:1)	1.077	2.154	0.097
>Stearic acid (C18:0)	1.077	1.077	0.393
>Oleic acid (C18:1)	1.077	2.154	1.391
>Linoleic acid (C18:2)	1.077	2.154	1.651
>Linolenic acid (C18:3)	1.077	2.154	1.504
>Ergosterol	1.077	1.077	0.992
>Total saturated fatty acids (SFAs)	3.232	3.232	2.983
>Total monounsaturated fatty acids (MUFAs)	2.154	4.309	1.489
>Total polyunsaturated fatty acids (PUFAs)	2.154	4.309	3.155
>Total lipid content	8.618	12.927	8.618

*SFAs-C14:0+C16:0+C18:0; MUFAs-C16:1+C18:1; PUFAs (C18:2+C18:3).*

### Sampling and Chemical Analyses for Anaerobic Fermentations Supplemented With Lipids

Yeast fermentation rate (carbon dioxide production) was monitored throughout fermentation *via* weight loss. Samples were harvested when *S. cerevisiae* fermentations became complete (this will be termed “end of alcoholic fermentation” for all strains in this study). The yeast population at the end of fermentation was determined using a Counter ZB-2 (Beckman Coulter, Villepinte, France). Residual sugars, ethanol, glycerol, and fermentation derived acids were also analyzed when fermentations were terminated using High Performance Liquid Chromatography or HPLC (HPLC 1290 Infinity, Agilent Technologies, California, United States) with a Phenomenex Rezex ROA column (Agilent Technologies California, United States). Sulphuric acid or H_2_SO_4_ (0.005 mol/l, Merck, France) was used as a mobile phase with a flow rate of 0.6 ml/min at 60°C. The EZChrom software package was used for data analysis (Seguinot et al., 2020).

### Sampling and Lipid Analysis for Self-Evolving Fermentations With No Lipids

Similar to the previous fermentation, yeast performance was also monitored *via* weight loss. Samples were harvested when *S. cerevisiae* completed fermentation (also termed “end of alcoholic fermentation” for all strains in this study) and centrifuged at 4,193 *g*. The supernatant was discarded and the yeast pellet was stored at −80°C. Fatty acids and sterols were extracted from the yeast pellet using the protocol described by Williams et al. (2021).

### Statistical Analyses

Fermentations were performed in triplicate (with the exception of *K. marxianus* in Medium A) and the impact of lipid mixtures on fermentation parameters, primary metabolite production and yield was evaluated by using ANOVA in XLSTAT (*p* < 0.05). A Bonferroni corrected test with a modified significance level of 0.017 showed differences in some lipid treatments. The cellular fatty acid and sterol profile of yeasts was also analyzed using ANOVA and the significance was also corrected *via* a Bonferroni test. Furthermore, the cellular lipid profile of yeasts was analyzed using Principal Component Analysis (XLSTAT). A hierarchical cluster analysis was performed to analyze different groups of primary fermentation metabolite yields in XLSTAT.

## Results

### The Influence of Lipids on Fermentation Kinetics

**FIGURE 1:**
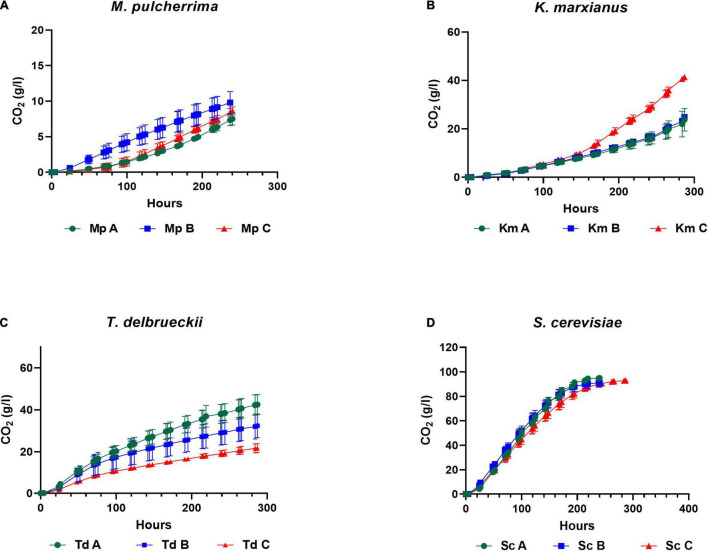
Carbon dioxide production (weight loss) in *M. pulcherrima* **(A)**, *K. marxianus* **(B)**, *T. delbrueckii* **(C)** and *S. cerevisiae* **(D)** in Medium A (green), Medium B (blue) and Medium C (red).

**FIGURE 2:**
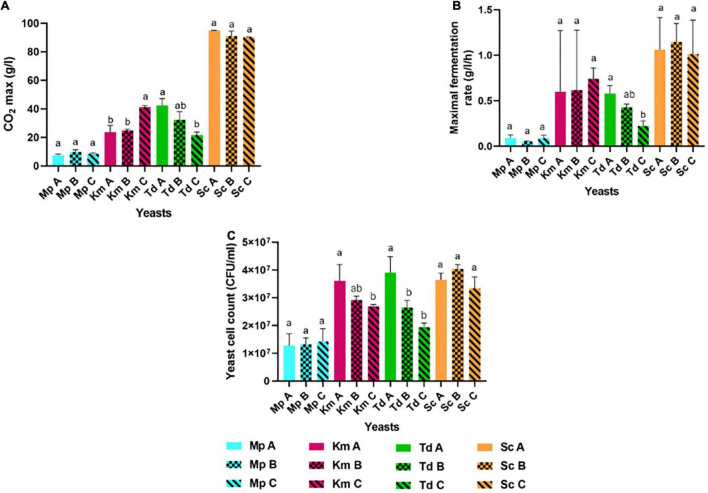
Maximal carbon dioxide or CO_2_ max production **(A)**, maximal fermentation rate **(B)** and yeast cell count **(C)** in *M. pulcherrima*, *K. marxianus*, *T. delbrueckii* and *S. cerevisiae* in Medium A, Medium B and Medium C. Blue bars-*M. pulcherrima* in Medium A (Mp A), B (Mp B) and C (Mp C). Pink bars—*K. marxianus* in Medium A (Km A), B (Km B) and C (Km C). Green bars—*T. delbrueckii* in Medium A (Td A), B (Td B) and C (Td C). Orange bars—*S. cerevisiae* in Medium A (Sc A), B (Sc B) and C (Sc C). Results obtained for treatments with the same letters are not statistically significant (*p* < 0.05).

Yeasts were pre-cultured in YNB medium for 18 h under semi-aerobic conditions (with 70% headspace) and harvested at the late exponential growth phase for inoculation into three synthetic grape juice-like media differing only in lipid composition (Table 2): Medium A contained equimolar concentrations of lipids, Medium B had double the amount of unsaturated fatty acids and Medium C contained a lipid mixture reflecting averages reported for natural grape juice (Yunoki et al., 2005; Tumanov et al., 2015). Medium B was used to determine whether the presence of higher amounts of unsaturated fatty acids improves fermentation performance compared to Medium A. On the other hand, Medium C was used to determine how the different yeast species perform in grape juice-like conditions based on averages reported for natural grape juice (Yunoki et al., 2005; Tumanov et al., 2015) when compared to the other two media. Fermentations were terminated when *S. cerevisiae* completed alcoholic fermentation (termed “end of alcoholic fermentation” for all strains in this study). The influence of the lipid treatments on yeast fermentation rate and yeast population dynamics are shown in Figures 1, 2. Overall and regardless of the lipid treatments, the lowest yeast population and carbon dioxide production was observed in *M. pulcherrima* followed by *K. marxianus*, *T. delbrueckii* and *S. cerevisiae* (Figures 1, 2). However, lipid ratios impacted yeast performances in a strain-specific manner. Fermentations in Medium C led to a significantly higher carbon dioxide production in *K. marxianus* at the end of alcoholic fermentation whereas maximal carbon dioxide production was significantly higher in Medium A when compared to Medium C in *T. delbrueckii*. Although not significant, *M. pulcherrima* displayed a slightly higher maximal carbon dioxide production in Medium B. The maximal fermentation rate was also higher in Medium C and A for *K. marxianus* and *T. delbrueckii*, respectively (although not significantly for *K. marxianus*). Interestingly, the yeast cell count was significantly higher in Medium A when compared to Medium C in *K. marxianus* and *T. delbrueckii* whereas no significant differences were observed in *M. pulcherrima*. In this study, minimal differences were observed in the yeast cell count, carbon dioxide production and fermentation rate between lipid treatments in *S. cerevisiae*.

### Lipids Impact Yeast Sugar Utilization and Metabolite Production

**FIGURE 3:**
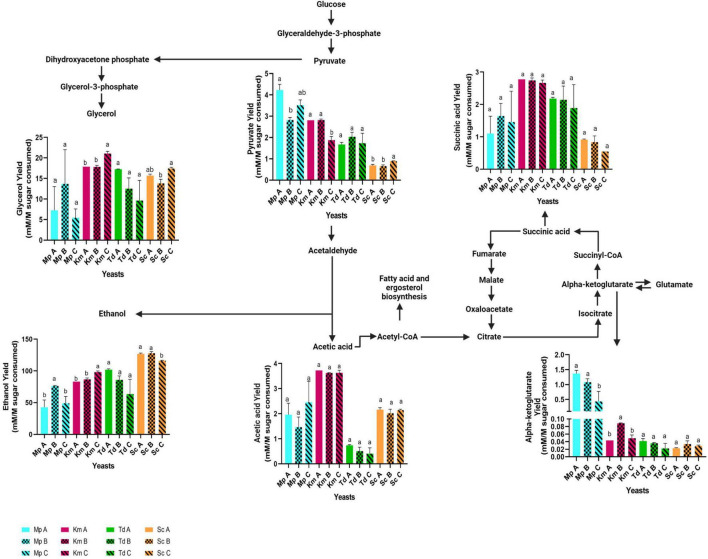
Fermentation derived metabolites yields in media containing different lipid combinations. Samples were harvested when fermentations were terminated and yields were calculated by normalizing the metabolites produced by sugar consumption (mM/M sugar consumed). Blue bars-*M. pulcherrima* in Medium A (Mp A), B (Mp B) and C (Mp C). Pink bars—*K. marxianus* in Medium A (Km A), B (Km B) and C (Km C). Green bars—*T. delbrueckii* in Medium A (Td A), B (Td B) and C (Td C). Orange bars—*S. cerevisiae* in Medium A (Sc A), B (Sc B) and C (Sc C). Results obtained for treatments with the same letters are not statistically significant (*p* < 0.05).

At the end of fermentation, residual sugars and primary metabolites were quantified (Table 3). Overall and regardless of lipid treatments, the highest residual sugar concentrations were observed in *M. pulcherrima*, followed by *K. marxianus*, *T. delbrueckii* and *S. cerevisiae* (which was the only strain to ferment to dryness) whereas the opposite trend was observed with ethanol. The highest glycerol concentrations were also observed in *S. cerevisiae*, followed by *K. marxianus*, *T. delbrueckii* and *M. pulcherrima*. Interestingly, *T. delbrueckii* produced the lowest amount of acetic acid followed by *M. pulcherrima*, *K. marxianus* and *S. cerevisiae*. Although *M. pulcherrima* fermented poorly, the highest alpha-ketoglutarate levels were observed in this yeast regardless of lipid treatments followed by *S. cerevisiae*, *K. marxianus* and *T. delbrueckii*. The highest pyruvate concentrations were observed in *K. marxianus* followed by *M. pulcherrima*, *T. delbrueckii* and *S. cerevisiae*. The overall highest succinic acid concentration was also observed in *K. marxianus* followed by *T. delbrueckii*, *S. cerevisiae* and *M. pulcherrima*. In *S. cerevisiae*, the presence of different lipid mixtures did not significantly impact sugar consumption and metabolite production. On the other hand, differences were observed in the residual sugar and metabolite concentrations for non-*Saccharomyces* yeasts due to lipid additions. Indeed, in *T. delbrueckii*, the lowest residual sugar concentration was observed in Medium A (96 g/l) when compared to Medium B and C (with residual sugar concentrations of 117 and 114 g/l, respectively). The lower residual sugar concentration in Medium A resulted in significantly higher amounts of ethanol, glycerol, α-ketoglutarate and acetic acid when compared to Medium C for *T. delbrueckii*. In *K. marxianus*, the lowest residual sugar concentration was observed in Medium C (96 g/l) when compared to Medium A and B (with residual sugar concentrations of 129 and 128 g/l, respectively). Consequently, ethanol, glycerol, acetic acid and succinic acid production was significantly higher in Medium C for this yeast. Compared to the other two lipid treatments, the residual sugar concentration was significantly lower in Medium B (with 132 g/l) for *M. pulcherrima* when compared to Medium A and C (with a residual sugar concentration of 155 and 151 g/l, respectively) resulting in a significantly higher ethanol concentration. Although the glycerol, acetic acid and succinate concentration was higher in Medium B for *M. pulcherrima*, it was not significant. On the other hand, the α-ketoglutarate concentration and pyruvate concentrations were significantly higher in Medium B for this yeast. Since fermentations were terminated at different stages, the concentration of metabolites deriving from central carbon metabolism and the Krebs cycle were normalized against sugar consumption (Figure 3). The pyruvate yield was significantly lower in media that resulted in the highest carbon dioxide production in *M. pulcherrima* and *K. marxianus* (Mp A and Km C) and the low pyruvate yields were linked to a significantly higher ethanol yield in these yeasts. Although acetate and succinate yields were highest in *K. marxianus* no significant differences were observed between lipid treatments. Furthermore, the lipid mixtures used in this study did not significantly impact acetic and succinic acid yields in the other yeasts. The highest α-ketoglutarate yield was observed in *M. pulcherrima* and some differences were observed between lipid treatments. In particular, the α-ketoglutarate yield was significantly higher in Medium A for *M. pulcherrima* when compared to Medium C. On the other hand, fermentations in Medium B resulted in a significantly higher α-ketoglutarate yield for *K. marxianus.*

**TABLE 3:** Residual sugars and fermentation derived metabolite production in Medium A containing an equimolar lipid composition, Medium B with double the amount of unsaturated fatty acids and Medium C with a grape juice-like lipid composition when fermentations were terminated (g/l).

Fermentations	Residual sugars	Ethanol	Glycerol	Acetic acid	Alpha-ketoglutarate	Pyruvate	Succinic acid
Mp A	155 ± 2a	7 ± 2b	0.623 ± 0.486a	0.265 ± 0.051a	0.076 ± 0.003ab	0.392 ± 0.009ab	0.075 ± 0.034a
Mp B	**131 ± 5b**	**20 ± 1a**	1.010 ± 0.121a	0.305 ± 0.104a	**0.092 ± 0.015a**	**0.395 ± 0.014a**	0.173 ± 0.049a
Mp C	151 ± 3a	9 ± 2b	0.515 ± 0.189a	0.355 ± 0.109a	**0.028 ± 0.023b**	**0.353 ± 0.003b**	0.105 ± 0.069a
Km A	129 ± 0a	23 ± 0b	2.485 ± 0b	0.7942 ± 0b	**0.004 ± 0b**	0.4092 ± 0a	0.301 ± 0b
Km B	128 ± 2a	24 ± 1b	2.500 ± 0.084b	0.780 ± 0.012b	**0.008 ± 0.0001a**	0.413 ± 0.004a	0.300 ± 0.009b
Km C	**96 ± 3b**	**40 ± 3a**	**4.274 ± 0.220a**	**1.129 ± 0.050a**	0.006 ± 0.0009ab	0.397 ± 0.024a	**0.421 ± 0.001a**
Td A	95 ± 6a	**42 ± 3a**	**3.525 ± 0.199a**	**0.231 ± 0.004a**	**0.005 ± 0.0006a**	0.358 ± 0.001a	0.347 ± 0.012a
Td B	116 ± 8a	28 ± 2ab	2.029 ± 0.36ab	0.125 ± 0.036ab	0.004 ± 0.0004ab	0.345 ± 0.007a	0.270 ± 0.036a
Td C	114 ± 4a	**21 ± 0.3b**	**1.544 ± 0.659b**	**0.098 ± 0.008b**	**0.002 ± 0.0006b**	0.295 ± 0.003a	0.237 ± 0.028a
Sc A	0.3 ± 0.02a	99 ± 1a	6.127 ± 0.140a	1.295 ± 0.045a	0.006 ± 0.0003a	0.284 ± 0.018a	0.278 ± 0.006a
Sc B	0.3 ± 0.1a	99.8 ± 2a	**5.389 ± 0.394b**	1.207 ± 0.097a	0.008 ± 0.002a	0.269 ± 0.024a	0.255 ± 0.059a
Sc C	0.3 ± 0.04a	98 ± 0.1a	5.664 ± 0.448a	1.282 ± 0.093a	0.007 ± 0.002a	0.235 ± 0.011a	0.239 ± 0.032a

*Results obtained for treatments with the same letters are not statistically significant and those in bold are significantly different (p < 0.05).*

### Evaluating the Impact of Lipids on Metabolite Yields Using Hierarchical Cluster Analysis

**FIGURE 4:**
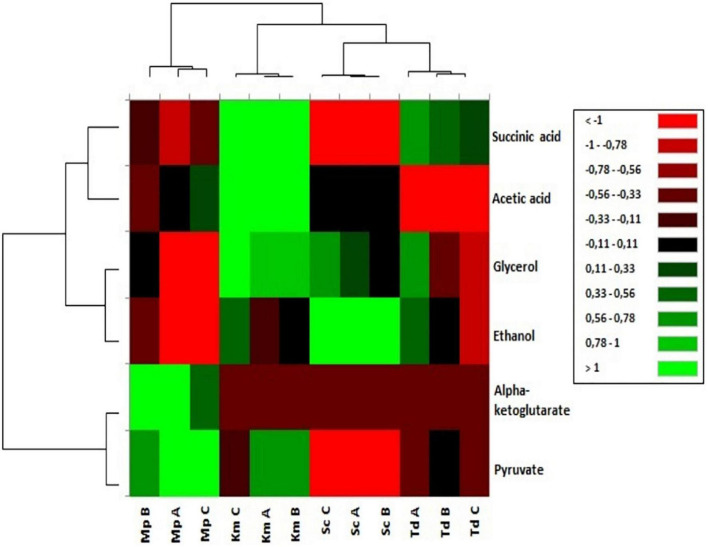
Hierarchical cluster analysis of fermentation derived metabolites yields (mM/M sugar consumed) grouped based on chemical properties and yeasts.

Fermentation-derived metabolite yields in synthetic grape-juice with different lipid mixtures were visually evaluated using Hierarchical Cluster Analysis (HCA) as shown in Figure 4. The relative abundance of metabolites was group dependent. Regardless of lipid mixtures, *M. pulcherrima* fermentations were characterized by a higher abundance of alpha-ketoglutarate and pyruvate. An increase in the relative abundance of acetic acid and succinic acid was observed for *K. marxianus* fermentations. *T. delbrueckii* fermentations were characterized by a strong reduction in the relative abundance of acetic acid whereas the relative abundance of ethanol was strongly enhanced in *S. cerevisiae*. For non-*Saccharomyces* yeast in this study, the relative abundance of ethanol and glycerol increased in media that resulted in the highest carbon dioxide production (Medium B for *M. pulcherrima*, Medium C for *K. marxianus* and Medium A for *T. delbrueckii*) but the enhancement in the relative abundance of these metabolites varied between strains.

### Yeast Lipid Composition in Synthetic Grape Juice-Like Medium With No Fatty Acids and Sterols

**FIGURE 5:**
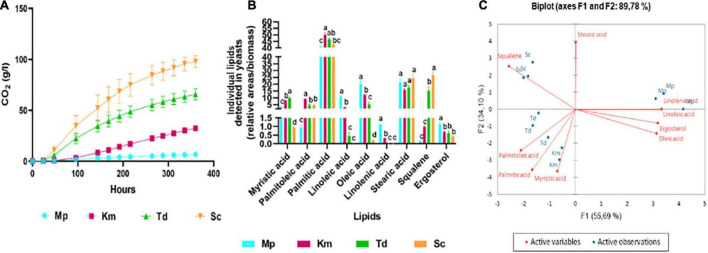
The carbon dioxide production **(A)**, cellular lipid profile **(B)** and principal component analysis biplot **(C)** of *M. pulcherrima, K. marxianus*, *T. delbrueckii* and *S. cerevisiae* at the end of alcoholic fermentation in synthetic grape juice-like media with no lipids. Blue bars-*M. pulcherrima*, pink bars—*K. marxianus*, green bars—*T. delbrueckii* and orange bars—*S. cerevisiae*. Results obtained for treatments with the same letters are not statistically significant (*p* < 0.05).

In order to better understand the impact of the three lipid mixtures reported above, the lipid composition of the four yeast strains was determined. Yeasts were inoculated into synthetic grape juice-like medium with no exogenous lipids and self-anaerobic fermentations were performed. Following the trends displayed above, the highest carbon dioxide production was observed in *S. cerevisiae* followed by *T. delbrueckii*, *K. marxianus* and *M. pulcherrima* (Figure 5A). Samples were harvested at the end of fermentation for cellular lipid analysis as shown in Figure 5B. Overall, stearic and palmitic acid were the main fatty acids present in all yeasts at the end of alcoholic fermentation, with very little differences between species. However, some differences were observed for the other lipids. For instance, *M. pulcherrima* produced significantly higher levels of linolenic, linoleic and oleic acids. On the other hand, palmitoleic acid level was significantly higher in *K. marxianus* while *T. delbrueckii* and *S. cerevisiae* produced significantly higher amounts of squalene when fermentations were terminated. The fatty acid and sterol profile of yeasts was visually evaluated using Principal Component Analysis (PCA) as shown in Figure 5C. Factor 1, representing 55.7% of the total variance, showed positive loadings for stearic acid, ergosterol, oleic, linoleic and linolenic acid whereas the opposite was observed for squalene, palmitoleic, palmitic and myristic acid. Factor 2, representing 34.1% of the variance, showed a positive loading for squalene, stearic and linolenic acid. *S. cerevisiae* fermentations clustered separately from the non-*Saccharomyces* yeast fermentations due to high amounts of squalene whereas *M. pulcherrima* clustered separately from the other yeasts due to the high levels of linoleic and linolenic acid. *K. marxianus* and *T. delbrueckii* clustered closely together due to palmitoleic, palmitic and myristic acid.

## Discussion

This study evaluated the impact of lipid mixtures on yeast performance during alcoholic fermentation. Regardless of the lipid treatment, the lowest carbon dioxide production was observed in *M. pulcherrima* followed by *K. marxianus*, *T. delbrueckii* and *S. cerevisiae* when fermentations were terminated. Similar to previous studies, *M. pulcherrima* fermented poorly (Shekhawat et al., 2017; Puškaš and Miljić, 2020) whereas *S. cerevisiae* fermented to completion (i.e., consumed all the sugars) leading to the highest ethanol, glycerol and acetic acid levels. On the other hand, *T. delbrueckii* produced moderate amounts of glycerol and the lowest acetic acid concentration, as previously reported (Renault et al., 2009; Mbuyane et al., 2018; Zhang et al., 2018) and the addition of different lipid mixtures did not alter the low acetic acid production trait of this yeast. This may indicate that acetic acid production in this yeast is impacted by other regulatory mechanisms that are not related to lipid metabolism or that the lipid concentrations used in this study did not significantly impact acetyl-CoA metabolism in *T. delbrueckii*. No significant differences were observed in *S. cerevisiae* following the addition of different lipid treatments. Interestingly, in a previous study, *S. cerevisiae* EC1118 was observed to be less sensitive to changes in the concentration of palmitic, oleic, linoleic and linolenic acid mixtures when compared to another strain (Liu et al., 2020). Similarly, this yeast may be less sensitive to the lipid concentrations and mixtures used in this study but may respond to higher lipid concentrations under more stringent fermentation conditions (i.e., higher sugar concentrations). So, the results obtained in this study for strain EC1118 may not be generalized to all strains of the species *S. cerevisiae*. Furthermore, fermentation conditions (such as the sugar concentration, oxygen conditions and type of lipid/s used) may cause discrepancies as previous studies have reported differences in EC1118 fermentation rate and metabolite production in response to different lipid mixtures (Duan et al., 2015; Liu et al., 2019).

### Species-Specific Impact of Lipids on Yeast Performance

This study shows for the first time that the addition of lipid mixtures impacts non-*Saccharomyces* yeast fermentation performance in a species-dependent manner, to a much greater extent than *S. cerevisiae*. Indeed, the fermentation of the three non-*Saccharomyces* yeasts investigated were improved when exposed to specific lipid mixtures. Although still fermenting poorly compared to the other species, *M. pulcherrima* performed somewhat better in the medium containing the highest level of unsaturated fatty acids and the data in this study also showed *M. pulcherrima* accumulated higher levels of unsaturated fatty acids in synthetic grape juice media with no lipids when compared to other yeasts. This indicates that this strain may have a higher demand for lipids innately found in the plasma membrane (i.e., unsaturated fatty acids) in an attempt to maintain membrane balance under conditions of stress. Indeed, since the accumulation of ethanol affects plasma membrane integrity, denatures important enzymes responsible for glycolysis and may affect nutrient uptake (Santos et al., 2008; Ma and Liu, 2010), the presence of unsaturated fatty acids in Medium B may have contributed to lowering ethanol sensitivity in this particularly sensitive species. Further studies are required to determine if *M. pulcherrima* has transport mechanisms different from *S. cerevisiae* which favor the uptake and/or utilization of unsaturated fatty acids. In *K. marxianus*, the highest fermentation rate was observed in Medium C with the highest concentration of palmitic acid and contained a higher total polyunsaturated fatty acid content (linoleic and linolenic) than the total monounsaturated fatty acid content (palmitoleic and oleic acid). Interestingly, *K. marxianus* produced the highest amount of palmitic acid and accumulated higher levels of monounsaturated fatty acids than polyunsaturated fatty acids when cultured in medium with no lipids. The data therefore suggests that the yeast lipid composition may determine the impact of exogenous fatty acids and sterols on yeast fermentation performance. However, further studies are required to confirm this observation in different strains. The highest carbon dioxide production *in T. delbrueckii* was observed in Medium A which contained an equimolar lipid mixture. Since *T. delbrueckii* accumulated intermediate concentrations of unsaturated fatty acids, this yeast may prefer media with a balanced lipid mixture. While the addition of unsaturated fatty acids was observed to increase ethanol shock resistance in *T. delbrueckii* in a previous study, (Pina et al., 2004) the polyunsaturated fatty acid cell content of *T. delbrueckii* was linked to ethanol sensitivity whereas the high ergosterol and monounsaturated fatty acid content of *S. cerevisiae* strains was linked to ethanol resistance (Aguilera et al., 2006) in another study. *T. delbrueckii* may therefore require media that contains all the necessary lipids required for anaerobic growth at an equimolar concentration in order to maintain plasma membrane structure/balance but further investigations are necessary to confirm this. Recently, it was observed that genes homologous to the sterol transporters *AUS1* and *PDR11* are absent in *T. delbrueckii*, *M. pulcherrima*, *K. marxianus* and other non-*Saccharomyces* yeasts (Dekker et al., 2021; Tesnière et al., 2021). Therefore, these yeasts may be more sensitive to changes in the fatty acid profile of the cell and may require specific fatty acid combinations for an improvement in fermentation performance especially under anaerobiosis.

### Lipids Impact Metabolite Production During Fermentation

This study also shows that the addition of specific lipid mixtures impacts the production of fermentation derived metabolites in non-*Saccharomyces* yeasts. An increase in the yeast fermentation rate led may lead to a higher utilization of pyruvate, an increase in ethanol and glycerol production (Erasmus et al., 2004; Varela et al., 2012; Goold et al., 2017; Casu et al., 2018). Although this was observed in all non-*Saccharomyces* yeasts, the increase in ethanol and/or glycerol was not always significant indicating that yeasts may require higher lipid concentrations or the presence of outliers (caused by oxygen ingress which affects metabolite production) may have minimized the effects of lipid mixtures.

Acetic acid is produced *via* the pyruvate dehydrogenase bypass whereby pyruvate is converted into acetaldehyde by a decarboxylase followed by the formation of this acid in a reaction catalyzed by an aldehyde dehydrogenase (Remize and Andrieu, 2000; Pigeau and Inglis, 2005, 2007). The pyruvate dehydrogenase bypass is closely linked to lipid metabolism because the resulting acetic acid produced in this pathway can be converted into acetyl-CoA which is an important building block for lipid biosynthesis (Van den Berg and Steensma, 1995; Kozak et al., 2014; Seguinot et al., 2020). Indeed, the presence of lipids was observed to decrease acetic acid production due to a reduced need for the carbon flux to be directed toward acetyl-CoA for lipid production (Luparia et al., 2004; Ochando et al., 2017; Deroite et al., 2018; Liu et al., 2020). On the other hand, excess unsaturated fatty acid concentrations were observed to increase acetic acid production in *S. cerevisiae* (Fairbairn et al., 2019; Liu et al., 2019). In this study, the presence of exogenous fatty acids and ergosterol did not significantly impact the acetate yield potentially due to inadequate concentrations being utilized, poor growth (in the case of *M. pulcherrima*) or the conditions not being stringent enough to elicit a reaction (in the case of *S. cerevisiae*). The highest acetic acid yield was observed in *K. marxianus* regardless of lipid treatments, probably as a result of this yeast’s higher carbon flux toward acetyl-CoA biosynthesis (Sakihama et al., 2019). This further highlights that the concentration of lipid mixtures utilized in this study was perhaps not enough to fulfill the acetyl-CoA demand of *K. marxianus* necessary for biosynthetic reactions. *K. marxianus* also produced the highest succinate levels in this study possibly due to the high flux toward acetyl-CoA synthesis which may enter into the TCA cycle (Sakihama et al., 2019).

Following the formation of acetyl-CoA, the pyruvate dehydrogenase bypass ends with shuffling this building block into the mitochondria *via* a carnitine acetyl transferase for use in the TCA cycle (Van Rossum et al., 2016). The TCA pathway operates as two branches during alcoholic fermentation instead of one complete cycle. While both branches ultimately lead to succinate accumulation, the oxidative branch occurs *via* the formation of α -ketoglutarate whereas the reductive branch is active *via* fumarate formation. In *S. cerevisiae*, the enzymatic activity of the oxidative branch was reported to be dysfunctional or minimal under anaerobic conditions and functional when glutamate was used as the main source of nitrogen (Pronk et al., 1996; Rodrigues et al., 2012). On the other hand, the reductive branch is known to be active during alcoholic fermentation as indicated by the activity of fumarate reductase (Camarasa et al., 2003). Furthermore, the biosynthetic reactions of the TCA cycle are also known to recycle reducing equivalents whereby NAD^+^ is converted to NADH (Chidi et al., 2018). Alpha-ketoglutarate is also integral in yeast nitrogen metabolism as it is utilized in the first step of the Ehrlich pathway to produce glutamate using NH_3_ in an NADP^+^-dependent reaction catalyzed by glutamate dehydrogenase. Thereafter, glutamate is converted into glutamine using NH_3_ in a reaction catalyzed by glutamine synthase (Hirst and Richter, 2016). On the other hand, glutamine can also be converted back into glutamate and finally α -ketoglutarate for utilization in the TCA cycle in a series of reactions that require glutaminases, NAD^+^-dependent glutamate dehydrogenases and yield NH_3_ (Guillamo and Verrips, 2001). In this study, an inverse relationship between α -ketoglutarate and succinate biosynthesis was observed potentially due to a redox balance regulation in the three non-*Saccharomyces* yeasts. Furthermore, *M. pulcherrima* produced higher amounts of α -ketoglutarate and lower levels of succinate whereas the opposite was observed in the other yeast strains. This may indicate that this yeast regulates its biosynthetic reactions and redox balance using mechanisms different from the other yeasts. Alternatively, *M. pulcherrima* may require less amino acids (such as glutamate which is produced from α -ketoglutarate) than other yeasts in this study due to low growth. Thus, excess α -ketoglutarate would be excreted into the environment. Further investigations are necessary to determine α -ketoglutarate antioxidant properties in yeasts as this metabolite was reported to protect against lipid peroxidation (Bayliak et al., 2017).

## Conclusion

This study shows that while minimal differences were observed in the fermentation rate of the specific strain of *S. cerevisiae* investigated, significant differences were observed in the production of carbon dioxide in response to different lipid ratios in selected non-*Saccharomyces* yeasts (in particular, *K. marxiianus*). However, the impact of different lipid ratios on the biosynthesis of fermentation-derived products was not always clear. This study shows that the yeast lipid profile is species-dependent and that the impact of exogenous lipids on yeast fermentation performance may be dependent on the yeast lipid composition. Moreover, the species-specific response to the different lipid mixtures could be linked to acetyl-CoA availability and the regulation of redox balance *via* the TCA cycle as well as acetyl-CoA metabolism. However, further investigations are required to evaluate the impact of exogenous lipids on a broader range of fermentation derived metabolites (such as medium chain fatty acids, ethyl and acetate esters) in order to understand non-*Saccharomyces* yeast lipid metabolism during winemaking. Although the addition of lipids may not result in complete fermentations in non-*Saccharomyces* yeasts, these compounds may increase the fermentation performance and ultimately their metabolic footprint in wine.

## Data Availability Statement

The original contributions presented in the study are included in the article/supplementary material, further inquiries can be directed to the corresponding author/s.

## Author Contributions

LM performed the experiments, analyzed the data, and wrote the manuscript. AB, CC, FB, and BD conceived the experiments, supervised the experimental work, assisted in the interpretation of the data, and edited the manuscript. AO-J provided inputs in the design of the experiments and discussion of the data. All authors have contributed and agreed to the submitted version of the manuscript.

## Conflict of Interest

AO-J was employed by Lallemand SAS. The remaining authors declare that the research was conducted in the absence of any commercial or financial relationships that could be construed as a potential conflict of interest.

## Publisher’s Note

All claims expressed in this article are solely those of the authors and do not necessarily represent those of their affiliated organizations, or those of the publisher, the editors and the reviewers. Any product that may be evaluated in this article, or claim that may be made by its manufacturer, is not guaranteed or endorsed by the publisher.
